# Prevalence of community-acquired bacteraemia in Guinea-Bissau: an observational study

**DOI:** 10.1186/s12879-014-0715-9

**Published:** 2014-12-20

**Authors:** Joakim Isendahl, Cristovão Manjuba, Amabelia Rodrigues, Weiping Xu, Birgitta Henriques-Normark, Christian G Giske, Pontus Nauclér

**Affiliations:** Department of Microbiology, Tumor and Cell Biology, Karolinska Institutet, Stockholm, Sweden; Department of Medicine Solna, Unit of Infectious Diseases, Karolinska Institutet, Stockholm, Sweden; Department of Paediatrics, Hospital Nacional Simão Mendes, Bissau, Guinea-Bissau; Bandim Health Project, Bissau, Guinea-Bissau; Department of Clinical Microbiology, Karolinska University Hospital, Stockholm, Sweden; Department of Infectious Diseases, Karolinska University Hospital, Stockholm, Sweden

**Keywords:** Bacteraemia, Sub-Saharan Africa, Guinea-Bissau, Antibiotic resistance, Malaria, Paediatrics, Molecular epidemiology

## Abstract

**Background:**

The burden of bloodstream infections is insufficiently studied in children in Africa and many healthcare facilities lack the capacity to identify invasive disease. Often studies have been limited to febrile patients or patients admitted to hospital.

**Methods:**

Blood cultures and malaria diagnostics was performed on 372 consecutive children presenting with tachycardia and/or fever to a referral paediatric emergency department in Bissau, Guinea-Bissau. Bacterial species detection, antimicrobial susceptibility testing and molecular typing were performed. The capacity of clinical parameters to identify bacteraemia was evaluated.

**Results:**

The prevalence of bloodstream infection was 12% (46/372) and in 46% (21/46) of the infections the child was non-febrile at presentation to the hospital. The predictive value for bacteraemia was poor for all assessed clinical parameters. *Staphylococcus aureus* accounted for 54% (26/48) of the isolates followed by non-typhoidal *Salmonella,* 10% (5/48), *Streptococcus pneumoniae,* 8% (4/48), and *Salmonella* Typhi, 6% (3/48). Among *S. aureus* there was a large diversity of *spa* types and 38% produced Pantone-Valentine leukocidin. Antibiotic resistance was low, however two out of three *Klebsiella pneumoniae* isolates produced extended-spectrum beta-lactamases. Malaria was laboratory confirmed in only 5% of the children but 64% (237/372) received a clinical malaria diagnosis.

**Conclusions:**

Bacteraemia was common irrespective of the presence of fever among children presenting to the hospital. The high prevalence of *Staphylococcus aureus* may be due to contamination. There is an imminent need to improve microbiological diagnostic facilities and to identify algorithms that can identify children at risk of bloodstream infections in Africa.

## Background

The burden of disease attributable to bloodstream infections (BSIs) is insufficiently studied in Africa where few healthcare facilities have the capacity to identify invasive diseases. BSIs are increasingly recognized as an important cause of hospitalisation and mortality in the region, and studies indicate that the prevalence of and mortality in bacteraemia parallels or exceeds that of malaria [1]-[4].

Due to symptom overlap bacteraemia is frequently misclassified as malaria and malaria is often over-diagnosed [3],[5]-[7]. Malaria may occur concomitantly with and has been indicated as a risk factor for bacteraemia [3],[8],[9]. Yet, the World Health Organization’s (WHO) guidelines for management of acute paediatric illness primarily focus on malaria and do not include BSIs in the diagnostic algorithm for fever in the absence of general danger signs [10],[11].

It would be valuable to identify children at risk of bacteraemia, where blood cultures should be performed. However, studies have demonstrated poor predictive values of clinical signs to detect bacteraemia [2],[4]. In a study from Kenya where blood cultures were performed on all children attending a hospital outpatient department, the sensitivity of fever to detect BSIs was only 73% [2].

There are reported differences in the aetiology of BSIs between African regions that may limit the effectiveness of treatment recommendations and immunization programmes [1],[12]-[16] and antibiotic resistance is an increasingly recognized problem [3],[4],[17]. There is to our knowledge no study that have investigated the prevalence of bacterial blood stream infections in Guinea-Bissau and many previous studies of bacteraemia in Africa have been limited to febrile or hospitalized patients, which may limit the generalizability of the findings [12]-[15].

The objective of this study was to investigate the prevalence, species distribution and antibiotic susceptibility of BSIs in consecutive children presenting with systemic infection signs to a paediatric emergency department in Guinea-Bissau. We also aimed to investigate the predictive capacity of clinical parameters to identify BSIs.

## Materials

### Study setting

The study was carried out between June and September 2010 at the paediatric emergency department of Simão Mendes, the national hospital in Guinea-Bissau. The department is the national referral centre for paediatrics, yet more than 90% of the enrolled children resided in or near the capital Bissau. The population of the capital is 423000 and 41% of the population is under age 15 [18]. The rainy season lasts from May-November. The community malaria prevalence in children was low in 2003 and 2008, 3% and 4% respectively [6],[19]. The adult HIV prevalence in 2006 was 4.6% for HIV-1 and 4.4% for HIV-2 [16]. Vaccinations against tuberculosis, polio, diphtheria, tetanus and pertussis have been performed since the 1980s (coverage 70-90% in 2009) while yellow fever, hepatitis B and *Haemophilus influenzae* type b vaccinations started in 2008–09 [20]. Blood cultures were not part of routine management at the paediatric department during the time of the study. Microscopic examination of blood for malaria was routinely available at the hospital at an extra fee.

### Study population

A pre-defined study size of 400 children was judged to be sufficient to provide an estimate of the burden of antibiotic resistance. The study was stopped when the pre-defined number of study participants was included. All children presenting to the emergency department between 9 am and 5 pm on weekdays were assessed for enrolment. The inclusion criteria were fever (axillary temperature ≥38°C) and/or tachycardia [<1 year ≥160 beats per minute (bpm), 1–5 years ≥120 bpm, measured with a pulse oximeter]. These criteria were selected since they together would have a high sensitivity to detect BSIs in children [2]. Two research nurses recorded clinical parameters, symptoms and clinical management. Inpatient mortality was retrieved from hospital registers. The exact number of children eligible for enrolment is not known, however the research nurses have declared that very few guardians declined participation for their child.

### Sampling and laboratory methods

Venous blood samples were drawn before the initiation of antibiotic treatment. The research nurses retrieved 3–4 ml (1 ml in neonates) of blood after thoroughly cleansing the skin with 70% ethanol. The samples were inserted into BactALERT Paediatric-fan blood culture bottles (bioMérieux, Marcy-l’Etoile, France), stored in ambient temperature overnight and then transported for incubation and culture at the National Public Health Laboratory. After 24 and 28 hours of incubation, the samples were cultured on in-house blood and chocolate agars and on a cysteine, lactose and electrolyte deficient (CLED) agar. Unique colony morphologies were frozen in −20°C in a freezing medium for sensitive bacteria used by and manufactured at the Department of Clinical Microbiology at Karolinska University Hospital in Stockholm, Sweden. At the end of the study period all samples were transported on dry ice to the Department of Clinical Microbiology at Karolinska University Hospital.

In Sweden, phenotypic species identification was performed with the VITEK2 system (bioMérieux). Antibiotic susceptibility patterns were established with the VITEK2 system, E-test (bioMérieux) and the disk diffusion method (Oxoid AB, Malmö, Sweden), using the standardised bacteriological methods, minimum inhibitory concentrations and breakpoints advised by the European Committee on Antibiotic Susceptibility Testing [21]. Blood cultures growing coagulase-negative *Staphylococci*, *Sphingomonas, Bacillus, Streptococcus viridans*, and *Acinetobacter* not confirmed as *Acinetobacter baumanii* were considered as probable contaminants and reported as negative in the analysis [4].

*Acinetobacter* isolates were subjected to the matrix-assisted laser desorption/ionization time-of-flight assay for species determination. Isolates producing extended-spectrum β-lactamases (ESBLs) were analysed with regard to resistance-encoding gene type with the Check-MDR multiplex PCR (Check-Points, Wageningen, The Netherlands). *Streptococcus pneumoniae* serotyping was performed by gel diffusion or capsular reaction testing [22]. *Staphylococcus aureus* isolates were characterized with regard to Protein A (*spa*) type and presence of Pantone-Valentine leukocidin (PVL) using an in-house method at the Swedish Institute for Communicable Disease Control.

Thin and thick smears for malaria microscopy were collected at admission to the hospital and subsequently read by an expert in Sweden. Droplets of blood were collected on filter paper from which DNA was extracted using the Chelex boiling method [23]. A nested PCR that targets the *cytb* gene (*cytb*-PCR), common to all *Plasmodium* species, was performed followed by a restriction fragment length polymorphism assay to determine parasite species [24],[25]. Parasite densities were measured with an 18Sq-PCR assay [26]. Children aged 1–5 years with a positive blood smear and/or *cytb*-PCR in conjunction with a parasite density ≥2500p/μl were considered to have clinical malaria. For children aged <1, a positive *cytb*-PCR in conjunction with a parasite density above 10p/μl was considered positive. These cut-offs were chosen as they have been reported to have sensitivities and specificities of 80% to identify clinical malaria [27]. The results from these malaria analyses were not available to the clinicians at the hospital.

### Statistical analysis

P-values were calculated with Fisher’s exact test and the χ2 test for categorical data as appropriate. Student’s t-test was used for continuous data. The sensitivity, specificity and predictive capacities of clinical parameters to detect bacteraemia were calculated.

### Ethics

Written informed consent was obtained from the parent or guardian and documented on the first page of the study questionnaire. Both the informed consent and the study procedures were approved by the National Health Ethics Committee in Guinea-Bissau (reference number 15/CNES/2010) and by the Regional Ethical Review Board in Stockholm, Sweden (reference number 2011/64-31/1).

## Results

### General characteristics of children in the study

Clinical data and blood samples were obtained from 414 children that fulfilled the inclusion criteria. During two weeks in July 2010, 39 consecutive children were excluded due to lack of freezing media for conservation of bacteria. Three further children were excluded since their isolates were not frozen according to protocol. Hence, 372 children were included in the analyses of which 48% (180) were admitted to hospital. Categorized by the inclusion criteria, 46% (172/372) had fever and tachycardia, 45% (167/372) had only tachycardia while 9% (33/372) had only fever. The mean age was 1.7 years and 44% (163/372) were females. Of the children from whom we obtained a report 2% (5/325) had been hospitalized during the month prior to enrolment.

### Bacteraemia prevalence, molecular epidemiology and antibiotic susceptibility

Probable pathogens were isolated from the blood of 12% (46/372) of the children (Table 1). *S. aureus* accounted for 54% (26/48) of the isolates while non-typhoidal *Salmonella* (NTS) accounted for 10% (5/48), *S. pneumoniae* for 8% (4/48) and *Salmonella* Typhi for 6% (3/48). Two children had polymicrobial bacteraemia, one with *S. aureus* and *Streptococcus pyogenes,* and one with *S. aureus* and *S. pneumoniae.* Three out of five children under 60 days of age were infected with Enterobacteriaceae. Gram-negative bacteria caused 55% (6/11) of the BSIs in children aged <1 year, compared to 24% (9/37) in children aged 1–5 years (p = 0.07). Among hospitalized children 14% (25/180) had bacteraemia, compared to 11% (21/192) among outpatients (p = 0.40). *S. aureus* may be part of the skin flora and hence it is possible that some of these findings were due to contamination. The corresponding figure for non-Staphylococcal bacteraemia was 8% (14/180) in the hospitalized group compared to 4% (8/191) of the outpatients (p = 0.14). There was no mortality among hospitalized children with Staphylococcal bacteremia (0/11), compared with 14% (2/14) in children with non-Staphylococcal bacteraemia (p = 0.49). The two fatalities in the bacteraemia group were diagnosed with neonatal sepsis and the isolated pathogens were *Escherichia coli* and *Enterobacter cloacae.*

**Table 1:** **Blood culture findings from children in Guinea-Bissau in relation to age and hospitalization**

Pathogen	Total (col. %)	Age stratified prevalence (col. %)				Hospitalized (col. %)	
		0-60 days	>60-365 days	>1-2 years	>2-5 years	Yes	No
	N = 372	N = 22	N = 79	N = 118	N = 105	N = 180	N = 191
Gram-positive	33 (9)	2 (7)	3 (4)	12 (9)	16 (13)	18 (5)	15 (4)
*Staphylococcus aureus*	26 (7)	2 (7)	3 (4)	10 (7)	11 (9)	13 (3)	13 (3)
*Streptococcus pneumoniae*	4 (1)	**-**	**-**	**-**	4 (3)	4 (1)	**-**
*Streptococcus pyogenes*	1 (0)	**-**	**-**	**-**	1 (1)	1 (0)	**-**
*Enterococcus faecalis*	2 (1)	**-**	**-**	2 (1)	**-**	**-**	2 (1)
Gram-negative	15 (4)	3 (11)	3 (4)	4 (3)	5 (4)	9 (2)	6 (2)
*Klebsiella pneumoniae*	3 (1)	**-**	2 (2)	**-**	1 (1)	2 (1)	1 (0)
*Escherichia coli*	2 (1)	2 (7)	**-**	**-**	**-**	1 (0)	1 (0)
*Salmonella typhi*	3 (1)	**-**	**-**	**-**	3 (2)	2 (1)	1 (0)
Non-typhoidal *Salmonella*	5 (1)	**-**	1 (1)	3 (2)	1 (1)	2 (1)	3 (1)
*Enterobacter cloacae*	2 (1)	1 (4)	**-**	1 (1)	**-**	2 (1)	**-**
Total children positive	46 (12)	5 (23)	6 (8)	16 (14)	21 (20)	27 (8)	21 (6)

Probable contaminants were isolated in 31% (117/372) of the samples. Prevalent contaminants were coagulase-negative *Staphylococci* (n = 87), *Sphingomonas* (n = 4), *Bacillus* (n = 4), and viridans group *Streptococci* (n = 3). None out of four *Acinetobacter* was confirmed as *Acinetobacter baumanii* in MALDI-TOF analysis and they were hence regarded as contaminants. To assess if detection of *S. aureus* was associated with contaminants, the prevalence of contaminants was evaluated among patients with *S. aureus*, patients with other probable pathogens, and patients with no growth of probable pathogens. There was no difference in the frequency of contaminants, 5/26 (19%), 6/20 (30%), and 106/326 (33%), respectively (p = 0.39).

The prevalence of *S. aureus* was similar in children admitted to hospital (7%, 13/180) and outpatients (7%, 13/192) (p = 0.87). Frequent *spa* types were t084 (n = 7), t355 (n = 5), t127 (n = 2), t1476 (n = 2) and t4690 (n = 2). The *spa* types t008, t024, t314, t491, t571, t760, t939 and t1458 were found in one isolate, each. In total, 38% (10/26) of the isolates produced PVL. The four *S. pneumoniae* isolates belonged to serotypes 6B (n = 2), 5 (n = 1) and 23F (n = 1).

Antibiotic resistance was uncommon among Gram-positive isolates (Table 2). All *S. aureus* isolates remained susceptible to methicillin. Both *Enterococcus faecalis* isolates were susceptible to ampicillin and vancomycin. One out of four *S. pneumoniae* isolates had a minimum inhibitory concentration for penicillin of 0.5 mg/l. Among Gram-negative isolates, two out of three *K. pneumoniae* isolates produced ESBLs (one TEM and the other SHV type). Similar to one of the two *E. cloacae* isolates, the ESBL-producing *K. pneumoniae* isolates were resistant to all tested antibiotics but imipenem. One of the patients with ESBL-producing *K. pneumoniae* had been hospitalized within the last month. The *Salmonella* had uniformly low minimum inhibitory concentrations to ciprofloxacin and azithromycin, indicating susceptibility.

**Table 2:** **Antibiotic resistance in bacteraemia isolates from children in Guinea-Bissau** ^**1**^

Species		Antibiotic drug, n (%)												
	***n*** (n)	AMP	AMC	PCG	CLX	CTX	TET	AZI	ERY	GEN	CLI	SXT	CIP	CHL
Gram-positive	33													
*Staphylococcus aureus*	26	-	-	-	0	0	2 (8)	-	0	0	0	0	0	0
*Streptococcus pneumoniae*	4	0	-	1 (25)^2^	-	0	3 (75)	-	0	-	0	4 (100)	-	3 (75)
*Streptococcus pyogenes*	1	0	-	0	0	0	0	-	0	-	0	0	-	1 (100)
*Enterococcus faecalis* ^3^	2	0	0	-	-	-	-	-	-	0	-	2 (100)^4^	-	1 (50)
Gram-negative	15													
*Escherichia coli*	2	1 (50)	0	-	-	0	-	-	-	0	-	1 (50)	0	0
*Klebsiella pneumoniae*	3	3 (100)	2 (67)	-	-	2 (67)	-	-	-	2 (67)	-	2 (67)	2 (67)	1 (33)
*Salmonella* Typhi	3	0	0	-	-	0	-	0	-	0	-	1 (33)	0	1 (33)
Non-typhoidal *Salmonella*	5	2 (40)	0	-	-	0	-	0	-	0	-	2 (40)	0	2 (40)
*Enterobacter cloacae* ^5^	2	-	2 (100)	-	-	1 (50)	-	-	-	1 (50)	-	1 (50)	1 (50)	2 (100)

AMP = Ampicillin, AMC = Amoxicillin-clavulanate, PCG = Penicillin G, CLX = Cloxacillin, CTX = Cefotaxime, TET = Tetracycline, AZI = Azithromycin, ERY = Erythromycin, GEN = Gentamicin, CLI = Clindamycin, SXT = Trimethoprim-sulfamethoxazole, CIP = Ciprofloxacin, CHL = Chloramphenicol.

^1^Defined as isolate tested R by EUCAST rules [21] if not otherwise stated.

^2^Intermediately resistant, minimum inhibitory concentration 0.5 mg/l.

^3^Both *E. faecalis* isolates were susceptible to vancomycin and linezolid.

^4^Intermediately resistant, inhibition zone diameters 30 and 24 mm respectively.

^5^Both *E. cloacae* isolates were susceptible to imipenem.

### Predictors of bacteraemia

In analysis of all 46 children with growth of a pathogen in their blood sample, no difference in age between children with and without bacteraemia was observed (p = 0.54). The bacteraemia prevalence was 10% (16/163) among females and 14% (30/209) among males (p = 0.19). Fever had 54% (25/46) sensitivity to detect bacteraemia and its positive predictive value (PPV) was only 12% (Table 3). Nearly all bacteraemic children (96%, 44/46) had tachycardia. However, the specificity of tachycardia as defined by our inclusion criteria was only 10% and the PPV was 13%. At higher tachycardia cut-offs, the specificity increased at the expense of reduced sensitivity while the PPV remained low. The other investigated clinical parameters were also poorly predictive of bacteraemia (Table 3).

**Table 3:** **Clinical predictors of bacteraemia in children in Guinea-Bissau with fever and/or tachycardia**

Clinical parameter		Factor present (n)						
	Missing obs. ^1^	Bacteraemia	No bacteraemia	P-value	Sensitivity	Specificity	PPV ^2^	NPV ^3^
		N = 46	N = 326		%	%	%	%
Temperature								
≥38°	-	25	181	0.88	54	44	12	87
≥39°	-	9	45	0.30	20	86	17	88
≥40°	-	3	6	0.09	7	98	33	88
Tachycardia^4^								
≥120 ≥ 160	-	44	295	0.25	96	10	13	94
≥130 ≥ 170	-	39	265	0.57	85	19	13	90
≥140 ≥ 180	-	23	167	0.88	50	49	12	87
Saturation^5^	4	3	10	0.20	7	97	23	88
Respiratory distress^6^	5	20	218	<0.01	44	32	8	81
Anaemia^7^	-	7	49	0.97	15	85	13	88
Convulsions	10	1	11	1.00	2	97	8	87
Lung crepitation	-	13	130	0.13	28	60	9	86
Diarrhoea	-	16	106	0.76	35	67	13	88
Reduced consciousness^8^	17	4	29	1.00	9	91	12	89
Vomiting	-	15	97	0.69	33	70	13	88
MUAC^9^								
<135 mm	-	9	78	0.51	23	72	10	87
<125 mm	-	3	35	0.60	8	87	8	87
<115 mm	-	0	12	0.37	0	96	0	87
Leukocyte count ^10^								
>11*10^9^/L	85	20	117	0.41	54	53	15	89
>20*10^9^/L	85	3	12	0.42	8	95	20	88
>11*10^9^/L and/or fever	85	29	183	0.56	78	27	14	89
Malaria positive	52	4	13	0.25	10	95	24	88

^1^Missing observations may pertain either to the bacteraemic or the non-bacteraemic group.

^2^Positive predictive value.

^3^Negative predictive value.

^4^Heart rate cut-off for tachycardia in children aged <1 year and 1–5 years, respectively.

^5^Oxygen saturation of haemoglobin <90%.

^6^Respiratory rate ≥40 per minute in children aged 1–5 years and ≥60 in children aged <1.

^7^Conjunctival pallor reported by the attending paediatrician.

^8^Defined as less than alert on the AVPU scale (A = alert, V = voice, P = pain, U = unconscious).

^9^Mid-upper arm circumference, measured on 317 children ≥6 months of age.

^10^Number of leukocytes per cubic millimeter.

Similarly, in a subgroup analysis of the 22 non-Staphylococcal bacteraemia cases we detected no difference in the prevalence of bacteraemia with regard to age (p = 0.32), sex (p = 0.47) nor the presence of fever (p = 0.21). There was weak evidence that fever of 39°C (p = 0.08) and a leukocyte particle concentration of ≥20 ×10^9^/l (p = 0.07) was more common in non-Staphylococcal bacteraemia while PPV was low at 11% and 20%, respectively.

We further assessed predictors of bacteraemia in the 167 non-febrile children (Table 4). All parameters had a poor trade-off between sensitivity and specificity. Fifteen non-febrile children had *S. aureus* infection, compared to 11 febrile children (p = 0.17). Also, *E. faecalis* (2)*,* ESBL-producing *K. pneumoniae* (2)*, E. coli* (1), NTS (1) and *S. pneumoniae* (1) were isolated from non-febrile children.

**Table 4:** **Clinical predictors of bacteraemia in non-febrile children in Guinea-Bissau**

Clinical parameter		Factor present (n)						
	Missing obs. ^1^	Bacteraemia	No bacteraemia	P-value	Sensitivity	Specificity	PPV ^2^	NPV ^3^
		N = 21	N = 145		%	%	%	%
Tachycardia^4^								
≥120 ≥ 160	-	21	145	N/A	100	0	13	N/A
≥130 ≥ 170	-	17	132	0.15	81	9	11	76
≥140 ≥ 180	-	10	76	0.68	48	48	12	86
Saturation^5^	1	1	3	0.42	5	98	25	88
Respiratory distress^6^	2	7	91	0.01	33	36	7	79
Anaemia^7^	-	2	18	1.00	10	87	10	88
Convulsions	5	1	0	0.13	5	100	100	88
Lung crepitation	-	6	55	0.41	29	62	10	86
Diarrhoea	-	10	55	0.40	48	62	15	89
Reduced consciousness^8^	10	1	3	0.43	5	98	25	88
Vomiting	-	7	46	0.88	33	68	13	88
MUAC^9^								
<135 mm	-	5	36	1.00	28	72	12	88
<125 mm	-	2	15	1.00	11	88	12	88
<115 mm	-	0	5	1.00	0	96	0	87
Leukocyte count^10^								
>11*10^9^/L	44	8	39	0.41	50	63	17	89
>20*10^9^/L	44	1	5	0.58	6	95	17	87
Malaria positive	23	1	1	0.24	6	99	50	88

^1^Missing observations may pertain either to the bacteraemic or the non-bacteraemic group.

^2^Positive predictive value.

^3^Negative predictive value.

^4^Heart rate cut off for tachycardia in children aged <1 year and 1–5 years, respectively.

^5^Oxygen saturation of haemoglobin <90%.

^6^Respiratory rate ≥40 per minute in children aged 1–5 years and ≥60 in children aged <1.

^7^Conjunctival pallor reported by the attending paediatrician.

^8^AVPU scale alertness level less than “awake”.

^9^Mid-upper arm circumference, measured on 149 children ≥6 months of age.

^10^Number of leukocytes per cubic millimeter.

### Clinical management

Empirical antibiotic therapy with adequate coverage of the isolated pathogen was prescribed to 61% (28/46) of the children. The treatment regimen was selected individually by the paediatrician, commonly ampicillin supplemented with a single dose of gentamicin. Out of the bacteraemic children, 22% (10/46) did not receive any antibiotic. Among the 18 children that received no or inadequate antibiotic treatment *S. aureus* caused 12 (67%) episodes and S. Typhi two, while *E. faecalis,* NTS, *E. cloacae* and *K. pneumoniae* caused one episode each. There was no difference in bacteraemia prevalence with regards to clinical diagnosis. Although the bacteraemia prevalence was similar in children with and without a clinical malaria diagnosis (p = 0.67), malaria-diagnosed children were less likely to receive antibiotic treatment (p = 0.002). Only 18% (5/28) of the malaria-diagnosed children with bacteraemia received adequate antibiotic treatment.

Malaria was diagnosed with microscopy in 13/227 (6%) children for which we obtained a slide and with PCR in 14/311 (5%) children for which we obtained a filter paper. In total, malaria was confirmed with either method in 5% (17/320) of the investigated children. In comparison, 64% (237/372) received a clinical malaria diagnosis by the attending physician. The bacteraemia prevalence in children with and without laboratory-confirmed malaria was 24% (4/17) and 12% (37/303), respectively (p = 0.25).

## Discussion

This is to our knowledge the first study on the prevalence of bacteraemia in children in Guinea-Bissau. The prevalence of bacteraemia was 12% in this paediatric population and malaria was over-diagnosed (found in 5% using laboratory tests, but in 64% using clinical criteria). As many as 46% of the children with bacteraemia did not have fever at presentation to the hospital and no clinical parameters predicted bacteraemia with high sensitivity and specificity.

The prevalence of bacteraemia in previous studies from Africa has ranged from 2% to 46% [1]-[4],[13],[14],[28], however most of these studies either did not use reproducible enrolment criteria [14],[28] or were focused on inpatients [1],[4],[17]. We observed a poor sensitivity of 54% for fever to detect bacteraemia. Excluding *S. aureus* bacteraemia the sensitivity improved slightly to 68% which is comparable to the 73% reported in a benchmark study by Brent *et al.* where children at a Kenyan outpatient department were blood cultured regardless of clinical presentation [2]. This implies that studies limited to febrile children may underestimate bacteraemia incidence and that the clinical threshold to perform blood cultures in children in Africa presenting to hospitals needs to be low. Similarly to the above mentioned study in Kenya, we found that tachycardia had higher sensitivity to detect bacteraemia but its specificity was poor [2]. In comparison, Nadjm *et al.* reported a 67% sensitivity of the current WHO criteria to detect invasive bacterial disease in a Tanzanian area of high malaria transmission [4]. Although our record of clinical parameters reflects the rudimentary conditions of paediatric care in Guinea-Bissau, neither clinical examinations nor prescribed treatments were harmonized with WHO recommendations. Therefore we could not evaluate the capacity of WHO criteria to predict bacteraemia. This may limit the generalizability of our analyses of clinical parameters in relation to bacteraemia. Future studies that assess the predictive capacity of clinical parameters and other screening tests need to assess these in relation to current WHO guidelines. Also, the epidemiological design needs to be carefully assessed to avoid selection bias both with regards to pathogen detection as well as in evaluation of screening algorithms.

More than half (54%) of the bacterial findings were *S. aureus,* which predominated also in studies from Malawi [12] and Nigeria [14],[28]. Several other studies of bacteraemia in Africa, including a systematic review, have reported *S. pneumoniae* [1]-[3] and NTS [4],[13],[17] as the most prevalent species (in our study the second and third most prevalent). In spite of similar findings in two previous studies from the region [14],[28] *S. aureus* may be part of the normal skin flora, and hence our finding of a high prevalence of *S. aureus* bacteraemia needs to be interpreted with caution. To investigate the pathogenicity of the *S. aureus* isolates they were subjected to *spa* typing. PVL-production has been reported to impair neutrophil function [29] and is associated with severe necrotizing pneumonia [30] and the high prevalence of PVL-production in our study is a concern. Furthermore, the *spa* type distribution closely resembled that of clinical isolates in a study from Gabon [31] and unlike studies from industrialized countries, types 084 and 355 (corresponding to MLST types 15 and 152) were the most prevalent.

The contamination frequency in previous studies of bacteremia in Africa has varied from 9-34% [1],[2],[8],[9],[12]-[15],[32], however some studies did not report the prevalence of contamination [4],[17],[28] and there is variability with regards to what species are considered pathogens. Viridans group *Streptococci*, *Citrobacter*, *Aeromonas*, *Cryptococcus and Morganella* are bacterial geni that may be considered pathogenic [12]-[14] but that we regarded as contaminants. Unlike previous studies [1],[2], we used MALDI-TOF to differentiate the often pathogenic *Acinetobacter baumanii* from low-pathogenic species*.* The wide range in prevalence of both bacteremia and contamination can thus partly be explained by differences in study design and reporting customs. In Guinea-Bissau blood cultures had not been routinely performed at the hospital or analyzed in the laboratory prior to our study, and the high frequency of contaminants implies that our protocol was not sufficient to avoid contamination. In future studies of bacteraemia in Guinea-Bissau, thorough training of hospital and laboratory staff is warranted. Also, since established as well as several new molecular methods are used in identifying microorganisms, epidemiological designs such as case–control studies are needed to obtain results on the clinical importance of microbe detection [33],[34].

Previous studies of invasive bacterial isolates in Africa have found varying prevalence of antibiotic resistance [3],[4],[12],[17]. In our study the overall antibiotic resistance was low, however two out of three *K. pneumoniae* produced ESBLs. We have recently reported that 33% of 408 children in Guinea-Bissau (from whom the 372 children in the present study originate) carried ESBL-producing Enterobacteriaceae in their intestines [35] and one of the two children with ESBL-producing *K. pneumoniae* bacteraemia in the present study was also a carrier. The presence of ESBLs also in bacteraemia is worrying considering the limited access to antibiotic drugs and the increased mortality in BSIs with ESBL-producing species [17]. The WHO’s empirical treatment recommendations for septicaemia is chloramphenicol and/or benzylpenicillin [10],[11]. The high prevalence of chloramphenicol-susceptible *S. aureus* in our material indicates this as an adequate treatment combination for suspected bacteraemia in Guinea-Bissau. Of the bacteraemia isolates in our study, 83% (40/48) were susceptible to at least one of these drugs.

There are limitations to our study. As discussed above the high prevalence of *S. aureus* may reflect contamination from the skin flora rather than infection. Interestingly however, removing *S. aureus* from the analysis did not alter our main finding that fever is an insufficient marker of bacteraemia. Since we did not measure CD4 counts or test for tuberculosis and other underlying conditions, we could not evaluate the influence of these parameters on the occurrence of bacteraemia. The study period coincided with the rainy season and was conducted at a national referral centre, which may have influenced the distribution of pathogens. Standardized laboratory testing with automated blood cultures and leukocyte counts would have strengthened the internal validity of our study. Finally we did not obtain follow-up data on outpatients, which hampered our analyses about mortality.

## Conclusion

In conclusion, the prevalence of paediatric bacteraemia in this study was high irrespective of the presence of fever. This indicates an urgent need to improve algorithms that can identify children at risk of bloodstream infections in Africa.
